# 30432 Novel insights from single-cell RNAseq analysis of the stromavascular fraction of human adipose tissue

**DOI:** 10.1017/cts.2021.413

**Published:** 2021-03-30

**Authors:** Bingyao Wang, Gregory Smith, Frederique Ruf-Zamojski, Gyu Ho Lee, Stuart C. Sealfon, Jeanine Albu, Susan K. Fried, Kalypso Karastergiou

**Affiliations:** 1 Icahn School of Medicine at Mount Sinai; 2 Department of Neurology at Icahn School of Medicine at Mount Sinai; 3 Diabetes, Obesity and Metabolism Institute at Icahn School of Medicine at Mount Sinai; 4 Obesity and Metabolism Institute at Icahn School of Medicine at Mount Sinai

## Abstract

ABSTRACT IMPACT: Characterizing the cellular composition of human adipose tissue may contribute to the prevention and/or treatment of obesity-associated metabolic diseases. OBJECTIVES/GOALS: Our aims in this study were to use single-cell techniques 1. to characterize cell types within the stromavascular fraction of human adipose tissue, 2. to identify subsets of cells within each type (sub-clustering), 3. to identify gene sets and pathways that may provide information on the function and significance of each cell cluster. METHODS/STUDY POPULATION: Abdominal subcutaneous adipose tissue samples from n=6 healthy volunteers (1M, 5F, age 28-38 y, BMI 24.5-63.0 kg/m2) were collected by aspiration or during surgery. In 3 subjects, all females, paired femoral samples were also collected. After collagenase digestion approximately n=10,000 cells/sample were used for single-cell RNA sequencing using the 10X Genomics platform. After QC and downstream analysis, data were analyzed in Seurat v.3.1.5. We identified first different cell types and then subclusters in an unbiased fashion. Gene Set Enrichment Analysis (GSEA) was used for pathway analysis. RESULTS/ANTICIPATED RESULTS: Progenitor markers are related to extracellular matrix, eg DCN (logFC progenitors vs other cells types=3.17, expressed in 99.7% (pct.1) of progenitors, 26.1% (pct.2) of others). Endothelial and pericytes shared markers like RBP7 (logFC=1.67, pct.1 0.88, pct.2 0.39); pericytes also showed unique markers, eg RGS5 (logFC=2.29, pct.1 0.89, pct.2 0.17). Progenitors are further divided into 11 sub-clusters, one of which showed enrichment of CD36 (high proliferation potential), FABP4 (differentiation), and of the novel marker PALMD (logFC=7.13, pct.1 0.94, pct.2 0.48). All p<10E-5. GSEA analysis suggests that inflammatory pathways are downregulated in both adipose progenitors and endothelial/pericyte cells in the femoral compared to the abdominal depot. DISCUSSION/SIGNIFICANCE OF FINDINGS: Single cell RNA sequencing provides unique insights into the molecular profile of cell types and the identification of novel subsets of cells within the human adipose tissue. Such cellular heterogeneity may explain differences in adipose function between individuals and eventually in the risk of obesity-associated metabolic diseases.

